# Collaboration Benefits All

**DOI:** 10.1200/JGO.19.00237

**Published:** 2020-01-10

**Authors:** Reda A. Hemida, Helena C. van Doorn, Leon F.A.G. Massuger

**Affiliations:** ^1^ Department of Obstetrics and Gynaecology, Mansoura University, Mansoura, Egypt; ^2^ Department of Gynaecologic Oncology, Erasmus MC Cancer Institute, University Medical Centre Rotterdam, Rotterdam, the Netherlands; ^3^ Department of Obstetrics and Gynaecology, Radboud University Medical Centre, Nijmegen, the Netherlands

Most women facing gynecologic cancer live in countries with inadequate health systems, and treatment is rarely in line with international standards. This is particularly true in cervical cancer, because most developing countries offer few opportunities for radiotherapy, leaving many women without proper treatment and the risk of avoidable mortality.^1^ The *Lancet* recently highlighted the need to close the global cancer divide for women, but real progress will require not only evidence-based policy making but also broad multisectoral collaboration and innovative public health approaches to cancer care and control.^2^ The gynecologic malignancy with the highest cure rate is gestational trophoblastic neoplasia (GTN). Postmolar GTN was studied at the Mansoura University Hospital, Mansoura, Egypt, with support from the Erasmus MC Cancer Institute, University Medical Center, Rotterdam, the Netherlands. The outcome of this study has implications for all women with GTN, and we would therefore like to share our experiences and at the same time advocate for an increase in worldwide partnerships of researchers to improve our understanding of routine clinical problems.

## Outcome of the Study From an International Perspective

In a randomized controlled trial, we studied the effects of second curettage in postmolar GTN on the number of methotrexate chemotherapy courses and found that a second curettage did not reduce the number of courses needed to normalize serum human chorionic gonadotropin (hCG) levels.^3^ Simultaneously, a multicenter prospective phase II study on the curative effect of recurettage in a similar patient group, performed in North America by the Gynecologic Oncology Group (GOG), showed that a second curettage cured 40% of women with postmolar GTN.^4^ Although the studies had different end points, we can conclude that second curettage is of value only when cure (ie, normalization of hCG) is expected. Together, the results of these studies finally settle the question of the role of second uterine curettage in postmolar GTN.

## Available Collaborations

After the formation of the Gynecologic Cancer InterGroup, the number of high-quality phase III trials at the global level has increased. However, most of the involved centers originate in developed countries. On meta-analysis of the global patterns of collaboration, most authors were found to be from larger centers in the northern and western United States and in Europe.^5^ Developing nations face many health challenges and lack the science, equipment, and financial infrastructure needed for research. Cooperation between the developing and the developed world is therefore mandatory.^6^ The benefits of collaborative projects extend well beyond scientific value alone. In this essay, we would like to emphasize several of these soft aspects.

## Some Studies Are Challenging in the Developed World

Certain studies cannot be efficiently carried out in developed countries because of low disease incidence and decentralized treatment; a case in point is GTN. The difficulty in patient accrual is reflected in our study^3^ and the GOG study^4^; in the GOG study, 9 centers recruited 64 patients over a period of 5.5 years, whereas the single-center Egyptian study recruited 89 patients in 4 years, indicating that international collaboration can sometimes overcome problems of slow patient recruitment.^6^

## Collaboration Improves the Standard of Care in Low-Income Countries

Health care benefits that may arise from contribution to clinical research include improvements in the local infrastructure, processes of care, and workforce.^7^ As a result of our involvement in the GTN study,^3^ practice at Mansoura Hospital has been evaluated and protocols updated in line with international standards. Other spinoffs of this collaboration include the launch of the first trophoblast clinic in Egypt, which now serves as a referral center for patients in the wider area, and the development in Arabic of a patient information leaflet on molar pregnancy. The trophoblast clinic has also established collaborations with sponsors to ensure that care remains available and is provided free of charge. Furthermore, this center recognized that recurrent mole (ie, ≥ 3 episodes of molar pregnancy) occurs more frequently than expected in the Egyptian Nile Delta (incidence figures not yet available). Moreover, collaboration with international experts was sought and chromosomal analyses performed, free of charge, in selected cases. This analysis resulted in the detection of mutations in several patients with recurrent molar pregnancies and led to the discovery of a recently published novel mutation.^8,9^

## Collaboration Improves the Standard of Research in Low-Income Countries

Through these alliances, colleagues who are unfamiliar with research practice are encouraged to adhere to international standards, beginning with a well-thought-out research protocol and proper recording of data, preferably using an electronic case record form. By engaging the resources of the supporting parties, such as Internet-based randomization programs and access to international literature, the research capacity of colleagues in a low-resource setting can be significantly enhanced. Collaborative analysis of data also allows for a better recognition of possible biases and weaknesses of a study. The knowledge obtained through hands-on support can be transferred to other researchers and staff members, further enhancing the positive effects of a collaborative study. In the case of this particular study, the sharing of results via a consensus workshop on gestational trophoblastic tumors has allowed us to reach gynecologists, pathologists, and oncologists all over Egypt. Finally, the presentation of results at international meetings and publication in high-impact journals benefit all researchers concerned.

## Collaboration Improves Mutual Understanding

Persistent GTN is one of the few neoplasias with a high cure rate, when treated appropriately. However, colleagues in low-resource countries often encounter problems unimaginable for professionals in the developed world. A range of challenges can frustrate effective treatment. For example, economic and political issues may obstruct patient travel to clinics for appointments, with resulting unwelcome delays in treatment. Some of these problems are gender related,^10^ whereas others require adaptations of research protocols to local situations.^11^ Women are more likely to be illiterate than men, and information given to the family may not always be shared with the patient. In many developing countries, women cannot or are not permitted by cultural norms to travel by themselves and therefore rely on a spouse or male family member to reach the hospital. Alternatively, women can be treated at home, an approach that might also be of value for women in the developed world, who at present have to visit a clinic for methotrexate injections 4 times within 8 days, repeated each cycle. Considerable time and expense might be spared if injections could be administered closer to home.

Given the many different national laws and regulations, performing international studies is challenging and costly. However, by performing similar, concurrent studies in multiple countries and uniting the outcomes of the various study sites, this disadvantage can be partly counterbalanced.^6,11,12^ Organizations such as the International Society for the Study of Trophoblastic Disease could take the lead in establishing these collaborations.

## Collaboration Is Fun

Last but not least, although international collaborations in which experienced researchers support colleagues in less fortunate circumstances certainly present many challenges, these partnerships can also be both valuable and enjoyable. Collaboration improves local care, offers professional satisfaction, and ultimately provides answers to shared scientific and clinical problems.
